# Biomarker research for Henoch-Schönlein purpura nephritis based on “omics” techniques

**DOI:** 10.3389/fmed.2025.1626324

**Published:** 2025-09-10

**Authors:** Xuejiao Zhou, Zhengwei Yuan, Hongkun Jiang, Yaoyao Ju, Cheng Chen, Wenjie Zhang, Huining Liu, Liqing Zhang, Meixia Qi, Dong An

**Affiliations:** ^1^Department of Pediatrics, The First Affiliated Hospital of China Medical University, Shenyang, China; ^2^Department of Pediatrics, The Fifth People’s Hospital of Datong, Datong, China; ^3^Key Laboratory of Health Ministry for Congenital Malformation, Shengjing Hospital, China Medical University, Shenyang, China

**Keywords:** Henoch-Schönlein purpura nephritis, immunoglobulin A vasculitis, biomarker, omics, Henoch-Schönlein purpura

## Abstract

Henoch-Schönlein purpura (HSP) is a common autoimmune disease in children. The lesions primarily involve small vessels in the skin, kidneys, joints and intestines. Henoch-Schönlein purpura nephritis (HSPN) caused by HSP is the main factor affecting the prognosis and outcome of the disease, and severe cases may develop into end-stage renal insufficiency. Renal biopsy serves as the primary diagnostic tool for HSPN, but it is an invasive test with poor reproducibility. Which is not conducive to clinical promotion. The discovery of new biomarkers using traditional laboratory testing methods and omics techniques provide new possibilities for HSPN to discover additional biomarkers, such as genomics, transcriptomics, proteomics, metabolomics and epigenomics. These technologies offer benefits such as high throughput, high sensitivity, and reduced need for biological fluid samples, which are expected to make greater contributions to the screening of promising biomarkers for purpuric nephritis. The current article reviews recent advances in omics-based research on potential biomarkers in samples from different sources of HSPN, including blood, urine, kidney tissue, and associated cells, which may provide a foundation for early diagnosis, prognosis, and treatment of HSPN.

## 1 Introduction

Henoch-Schönlein purpura (HSP), or immunoglobulin A vasculitis (IgAV), is a prevalent systemic vasculitis impacting the skin and various organs, especially small blood vessels in the joints, kidneys, and intestines. HSP is generally self-limiting, but it easily causes complications in children. When the kidneys are involved, it is called Henoch-Schönlein purpura nephritis (HSPN) or IgA vasculitis with nephritis (IgAVN). Children under the age of 10 are more frequently affected by HSP (1), and approximately 97% of them have kidney damage within 6 months of developing HSP, and children at an older age have a worse prognosis (2). A recent European study showed that the typical yearly occurrence of IgAV was 6.79 cases for every 100,000 people and the prevalence of IgAVN was 19.6% (3). A study from Japan estimated the yearly occurrence of biopsy-verified HSPN is 1.32 cases for every 100,000 individuals (4). Although most HSPN cases resolve spontaneously without treatment, there are some situations where patients may still end up with serious kidney failure despite receiving treatment, and the chance of this happening over time can be as high as 15% (5). Even children who are in remission 10 years after diagnosis develop chronic renal failure or other complications 20 years later (6). If renal function is severely impaired, dialysis or kidney transplantation is needed, which places a great burden on families and society. Therefore, continuous renal monitoring is necessary.

The commonly used diagnostic methods for HSPN include two types, invasive and non-invasive. Renal biopsy pathological examination is a common invasive examination in clinical practice. Immunofluorescence examination of the kidney demonstrated that the primary characteristic was the accumulation of IgA1-rich immune complexes in the kidney’s mesangial area, which was helpful for early diagnosis. The extent of kidney injury can be assessed through a kidney tissue examination to predict the outcomes of HSPN. The glomerular pathological grading method is widely used, and some studies believe that a proportion of crescents greater than 50% may indicate a poor prognosis (7). Some recent studies have used the MEST-C score (mesangial hypercellularity [M], endocapillary proliferation [E], segmental sclerosis/adhesion [S], tubular atrophy/interstitial fibrosis [T], and cellular or fibrocellular crescents [C]) for pathological grading and found that S and T may indicate poor prognosis of HSPN (8). M1 and T1/T2 scores show worse renal outcomes in children with HSPN compared to M0 and T0 scores (9). Another study found that patients without a kidney biopsy at the time of diagnosis needed anti-proteinuria therapy more than their peers who underwent a kidney biopsy (10). Therefore, a renal biopsy is crucial for diagnosing diseases early, assessing patient outcomes, and guiding treatment options. However, biopsy may lead to complications, such as hematuria, perirenal hematoma, and infection, due to its invasive nature. The examination also suffers from poor repeatability, high cost and poor patient compliance, which are not conducive to promotion in primary hospitals.

Non-invasive tests include biochemical tests of blood and urine. Immunoglobulin is one of the most studied laboratory indicators in recent years, but the results are different. IgA is the main immunoglobulins that fight infection. Many studies showed that serum galactose-deficient IgA1 (Gd-IgA1) was involved in the development of HSPN. In the HSP and HSPN groups, the amounts of serum IgA1 and Gd-IgA1 were greater than those in the control group, with the HSPN group showing much higher levels compared to the HSP group (11, 12). However, some studies showed there is no major difference in serum IgA levels when comparing the IgAV group to the IgAVN group, and higher serum IgA levels do not link to kidney issues related to IgAV (13). As a bridge mediating tissue damage and cellular immunity, the complement system also participates in the pathogenesis and development of HSPN. Although these indicators have important value in the early diagnosis and disease assessment of HSPN, they lack specificity. Therefore, they are primarily used for auxiliary diagnosis in clinical practice. Urine protein has become a routine detection index of HSPN in clinical practice, which is a marker of kidney damage and a key indicator of the progression of kidney disease (14). The pathological grade of HSPN positively correlated with 24-h urinary protein and microalbuminuria, and the level of albuminuria reflected the severity of renal injury of HSPN (15). Ye et al. found that the amount of protein in the urine over 24 h and the ratio of urinary protein to creatinine steadily rose as HSPN worsened (16). Collecting urine over a 24-h period is difficult and takes a lot of time. Factors like how concentrated the urine is, variations in sampling, and using preservatives can influence the results of the examination (17, 18). Serum creatinine (Scr) and blood urea nitrogen are the primary clinical measures utilized to evaluate how well the glomeruli filter blood, but they have many limitations. Scr may be affected by confounding factors, such as muscle content, fluid volume distribution, renal reserve function and glomerular hyperfiltration status (19). Some extrarenal factors may also affect urea nitrogen, and serum urea nitrogen is significantly elevated in the presence of gastrointestinal bleeding, severe infection, and insufficient effective blood volume (20). When the glomerular filtration rate (GFR) dropped below half of what is considered normal, there was a notable rise in levels of Scr and urea nitrogen. When Scr and blood urea nitrogen exceed the normal range, glomerular filtration function is significantly impaired. Therefore, Scr and urea nitrogen are not sensitive to the diagnosis of early renal injury (21).

In conclusion, traditional examination methods are not ideal in the early diagnosis and prognosis assessment of HSPN. It is essential to identify suitable biomarkers for the early detection of kidney damage in patients with HSP, as this will help direct their treatment and enhance their outlook. The present paper reviewed the research progress of purpuric nephritis biomarkers using omics techniques in recent years from the aspects of blood, urine, tissue and their related cells.

## 2 Omics screening of biomarkers in HSPN

Omics is a comprehensive technique for examining molecular interactions and relationships in biological phenotypes (22). The omics method performs a comprehensive analysis of some molecules, including genomics (DNA), transcriptomics (RNA), proteomics (proteins), metabolomics (metabolites), and epigenomics (23). Omics techniques are increasingly widely used in screening biomarkers. Compared to traditional test methods, these techniques have the advantages of fewer requirements for biological fluid samples, high sensitivity and high throughput. However, the application of omics techniques in the field of nephrology remains at an early stage. For example, it is primarily used in acute kidney injury (24), lupus nephritis (25), kidney transplantation (26), obstructive nephropathy (27, 28), immunoglobulin A nephropathy (29), HSPN, chronic kidney disease (30), and other kidney diseases. The advancement of technologies that can process large amounts of data quickly, along with extensive data gathering and computer-based analysis, has made it easier to find biomarkers for diagnosing diseases early and has helped us understand how diseases develop more thoroughly. For example, many research projects have shown that IgA1 with unusual glycosylation is crucial in the development of IgA vasculitis nephritis (IgAVN). In most patients with IgAV, IgA1 is missing galactose components (31). A genome-wide association study (GWAS) of adult patients with IgA nephropathy and elevated serum Gd-IgA1 levels identified two potential loci (C1GALT1 and C1GALT1C1) involved in the O-glycosylation pathway as possible causes of IgAV aberrant glycosylation (32). However, research based on omics techniques in HSPN remains in its early stages. We compiled an overview of the existing progress in finding biomarkers related to the diagnosis, therapy, and development of HSPN based on omics techniques. Omics technology is expected to make greater contributions in these aspects.

### 2.1 Genomics

Genomics is the analysis of nucleotide sequences and gene functions at the level of all genes in an organism. Applications of genomic technology include quantitative analysis (including real-time PCR) and high-throughput techniques (including microarrays and sequencing) (33). Several common genetic variants and polymorphisms are associated with the susceptibility and severity of HSPN. Most previous studies used PCR-based sequencing typing for genomics. Ren et al. used PCR-sequence-specific oligonucleotide analysis and PCR-sequencing-based typing (SBT) and discovered that HLA-A was a gene linked to HSP risk in blood samples from the Han population. HLA-A^*^11(^*^1101), HLA-B^*^15(^*^1501), HLA-B^*^35(^*^3503) and HLA-B^*^52 are related to the susceptibility of HSP in Mongolian and Han populations, and HLA-B^*^40 and HLA-B^*^07 may have a protective effect on HSP (34). Lopez-mejias and colleagues validated the link between HLA-DRB1 and HSP using PCR-sequence-specific-oligonucleotide probe (PCR-SSOP) in whole blood of a large Caucasian cohort. HLA-DRB1^*^01 was a marker of susceptibility to HSP, and HLA-DRB1^*^03 was protective against HSP (35). They also utilized SBT to show that the influence of HLA-B^*^41:02 on HSP vulnerability was not dependent on the status of HLA-DRB1^*^01:03. HSPN patients also carried this gene, but no statistical association was found (36). Soylemezoglu et al. also found that the CTLA-4 AG genotype and HLA-DRB1^*^13 may be risk factors for nephrotic proteinuria in patients with HSP using PCR-based DNA genotyping (37). Lopez-Mejias and colleagues showed that the IL1βrs16944 genetic variation could indicate serious kidney damage in HSP through the use of TaqMan genotyping (38).

Most past research regarding the genetic factors of HSPN has concentrated on specific genes through candidate gene studies. High-throughput sequencing technology identifies genome sequences related to diseases more quickly and accurately and identifies abnormal structural changes to determine pathogenic genes or susceptibility sites. An increasing number of studies have used various sequencing technologies to explore new gene polymorphisms and gene variants associated with HSPN. Xu et al. extracted DNA from peripheral blood and used matrix-assisted laser desorption/ionization time-of-flight (MALDI-TOF MS) to perform single nucleotide polymorphism (SNP) genotyping of the MMP-9 gene, and the results showed that the rs3918254 and rs17576 polymorphisms were associated with HSPN (39). Jiang et al. used the same method to genotype 10 functional SNPs of the inducible nitric oxide synthase (iNOS) gene in peripheral blood, and the results showed that the rs2297518 polymorphism may significantly impact the progression of HSP to HSPN (40). Chen and others utilized denaturing high-performance liquid chromatography (DHPLC) along with DNA sequencing to examine changes in the PAX2 gene. Their findings revealed that the occurrence of the PAX2 gene exon 8 variation (798C > T) was notably greater in the HSPN group compared to the HSP and healthy control groups. It may be used as a screening index for early renal injury in children with HSP (41). Lopez-Mejias et al. (42) performed the first GWAS for IgAV in a large European population cohort, and IgAV was a typical HLA Class II disease. Koskela and colleagues conducted a GWAS study using samples from blood and bone marrow donors. They discovered that the haplotype allele DQA1^*^01:01/ DQB1^*^05:01/ DRB1^*^ 01:01 was linked to a higher risk of developing HSP but did not have a correlation with the severity of kidney issues (43). Xia et al. also used GWAS to demonstrate that HLA-DRB1 was closely associated with IgAV susceptibility in blood samples from individuals in the Han Chinese population (44). Lee et al. identified a novel susceptibility locus (rs9428555) that was associated with the development of IgAV in Korean children using association testing and Sanger sequencing in blood samples (45).

However, there are many SNPs to be sequenced in GWAS, and the sequencing cost is high. With the gradual development of sequencing technology, next-generation sequencing (NGS, such as whole genome sequencing and whole exon sequencing) has become increasingly the most direct, rapid and accurate method to find genetic loci of diseases. Its main advantages are the small number of samples required and the ability to detect most variants, especially rare variants associated with disease (46). Ekinci and colleagues utilized next-generation sequencing to analyze the MEFV gene in 144 patients with HSP who did not show symptoms of Familial Mediterranean Fever. Their findings suggested that mutations in exon 10 of the MEFV gene could influence how HSP appears clinically (47). Jin et al. performed whole-exome sequencing on three DNA samples from an HSP family, then censored the associated single nucleotide variants (SNVs) and verified the variants using Sanger sequencing and PCR. Analysis of their results showed that immune-associated MIF and MGAT5 mutation sites may be associated with HSP susceptibility (48).

In conclusion, genomics is providing new insights into the genetic mechanisms associated with HSPN. Despite the stability and integrity of genomics and the non-invasive nature of genetic testing in blood, it has great potential in the diagnosis and monitoring of early renal injury in HSP. However, it is still constrained by sample size, ethnicity and other factors. The current studies were performed with small sample sizes, and large-scale analyses of these variants are needed in the future. An increasing number of studies used high-throughput sequencing technology. NGS may be a new opportunity to clarify the genetic mechanism of HSPN, search for specific biomarkers, and realize gene-targeted therapy in the future.

### 2.2 Transcriptomics

Transcriptomics involves examining gene transcripts, or RNA, that are produced by certain cells, tissues, or individuals at particular times. In contrast to genomics, which explores static DNA sequences, transcriptomics is used to assess dynamic gene expression (49). Previous studies mostly used microarray methods to find biomarkers. Levin et al. used microarray analysis of the dendrin gene in glomerular tissue of IgAN/IgAV patients and found higher dendrin mRNA levels in IgA-associated patients compared to controls. Patients with mild CKD had higher levels of dendrin protein gene expression, which may be a potential marker of IgA-associated nephropathy. However, they did not validate the microarray data with qPCR (50). Li et al. used cDNA microarray technology to screen the differential genes of HSP children in peripheral blood, and PCR was used to verify three of these genes. The findings indicated that the SCUBE1 gene was more active in the pure HSP group compared to the control group, and the keratin 18 gene had a greater expression in the HSPN group than in the basic HSP group. Additionally, the transglutaminase 2 gene showed less expression in the HSPN group than in the basic HSP group. The expression direction of each gene was consistent with the microarray results. These genes may be the susceptibility genes of HSP/HSPN (51). Over time, transcriptomic capabilities have evolved from traditional microarray technologies focusing only on mRNA to comprehensive analyses of all transcribed RNAs using RNA sequencing (RNA-seq) (52). A recent study examined the underlying mechanisms of HSPN by combining transcriptomic and proteomic analyses. Xie et al. used RNA-seq and tandem mass tag (TMT) quantitative proteomics techniques in the serum of HSPN patients, discovering that there were 2,315 mRNAs and 30 proteins that were expressed differently across various forms of HSPN. One protein and 58 mRNAs were identified as potential biomarkers for HSPN progression, and RPS17-201, which is consistently downregulated during disease progression, was considered the most promising marker. They selected ten differentially expressed mRNAs and ten proteins for verification and found that six mRNAs and six proteins were consistent with the omics data. They also identified four important pathways associated with disease progression using pathway enrichment analysis, suggesting that the occurrence of HSPN was related to the inhibition of inflammation, repair of renal injury and promotion of apoptosis (53) (Table 1).

**Table 1:** Omics techniques to identify biomarkers in the blood of HSPN patients.

Number of cases initially screened and sample types	Omics techniques	Methods	Validation biomarkers	Major related pathways	Reference
HSPN (*n* = 9) and HSP (*n* = 9)	Transcriptomics and proteomics	RNA-seq, TMT	FERMT3-201, CCND3-215, HNRNPK-204, SH3GL1–201, KIF2A-202, HSPA8-208, MGP, LPA, FCN1, CCL5, TGFB1, LTBP1	Negative regulation of the JAK–STAT cascade, the mTOR signaling pathway, the SWI/SNF superfamily type complex, and the Wnt signaling pathway	(53)
IgAV (*n* = 6), IgAVN (*n* = 6) healthy subjects (*n* = 7)	Proteomics	NanoLC–MS/MS, ELISA	SAA, KNG1, C4a, AGT	Homeostasis, Wnt signaling pathway	(68)
IgAV (*n* = 39), IgAVN (*n* = 6) and healthy controls (*n* = 6)	Metabolomics	Q-TOF LC/MS	No validation	Lysine degradation-*Homo sapiens* (human), Prostaglandin leukotriene metabolism, Prostaglandin formation from arachidonate, and Arachidonic acid metabolism	(84)
IgAV (*n* = 58) and healthy controls (*n* = 28)	Metabolomics	UPLC, LC–MS/MS	No validation	Cardiovascular disease, insulin resistance, cell apoptosis, and inflammation.	(87)
HSPN (*n* = 6) and healthy controls (*n* = 4)	Epigenomics	High-throughput sequencing, RT-qPCR	ENST00000378432, ENST00000571370, uc001kfc.1, uc010qna.2	p53 signaling pathway, apoptosis-associated genes (AKT serine/threonine kinase 2, tumor protein 53, phosphatase, tensin homolog and FAS).	(95)

HSPN, Henoch-Schönlein’s purpura nephritis; HSP, Henoch-Schönlein purpura; IgAV, IgA vasculitis; IgAVN, IgA vasculitis with nephritis; RNA-seq, RNA sequencing; TMT, tandem mass tag; LC–MS/MS, liquid chromatography–tandem mass spectrometry; DIA, data-independent acquisition; ELISA, enzyme-linked immunosorbent assay; SAA, serum amyloid A; KNG1, kininogen1; AGT, angiotensinogen; NanoLC–MS/MS, nanoscale ultraperformance liquid chromatography-mass spectrometry; Q-TOF LC/MS, Quadrupole time of flight mass spectrometry; UPLC, ultraperformance liquid chromatography; RT-qPCR, reverse transcription-quantitative polymerase chain reaction.

Although RNA-seq has been widely used in various fields of research, RNA-seq technology involves the bulk sequencing of RNA, which does not reveal underlying changes between cells. Therefore, single-cell sequencing (scRNA-seq) was first proposed in 2009 (54). Using scRNA-seq allows for the accurate detection of variations in gene activity among various cell types, as well as capturing the diversity and spatial arrangement of diseases. It can analyze gene activity and how cells communicate with each other, assess functional enrichment, explore metabolic pathways, and discover new cell subtypes in heterogeneous groups (55). ScRNA-seq technology has been widely used in kidney immunology to detect immune cells in blood, urine, lymphoid tissue or kidney biopsy tissue and discovered new immune cell populations and signaling (56). Numerous studies using single-cell sequencing that have identified potential markers and further elucidated the molecular mechanisms of IgAN (57–60). Notably, recent studies have employed single-cell sequencing technology to explore the pathogenesis of HSPN. Using single-cell sequencing technology, Ye et al. explored the potential connection between IgAN and IgAVN in kidney tissues. The results showed that the group with IgAN had a notably smaller percentage of proximal tubular (PT) cells than the group with IgAVN, with the downregulated DEGs primarily related to growth regulation and protein translation. Additionally, the expression levels of hypoxia-inducible factor (HIF1A) were significantly elevated in genes related to energy metabolism and fibrosis within the cells of pulmonary arterial hypertension in IgAN patients. Furthermore, in IgAVN patients, there was a significant increase in the expression of HIF1A target genes, including argininosuccinate synthetase 1 (ASS1). These genes play roles in several processes such as adapting to low oxygen levels, energy metabolism, programmed cell death, epithelial-mesenchymal transition (EMT), kidney repair, and tubular EMT. The study eventually found variations in the expression changes of the HIF1A gene and energy metabolism between the two diseases, which provided an important basis for discovering the association between IgAN and IgAV (61) (Table 2). Zhou et al. applied single-cell transcriptomics to explore the variations in immune cell composition in patients with different HSP types. The results showed an expansion of Tfh cells, B cells, and plasma cells in the joint and abdominal types. The skin and kidney types showed an enrichment of cytotoxic effector T cell subsets, thereby emphasizing the dynamic nature of the immune response during the different clinical manifestations of HSP (62). Liu and colleagues applied this approach to explore the ratio, characteristics, and role of mucosal-associated invariant T (MAIT) cells in groups with HSP and healthy individuals. The study results revealed a decreased proportion of MAIT cells in the HSP group, with an upregulation of genes related to T cell activation and effector functions, indicating a more active phenotype. Moreover, CD40L^+^ MAIT cells can stimulate the production of IL-4 and promote the production of IgA by B cells, highlighting the significant role of MAIT cells in disrupting homeostasis in HSP patients (63) (Table 2).

**Table 2:** Biomarkers of HSPN in urine, renal tissues or cells identified using omics.

Number of cases initially screened	Omics technique	Methods	Validation biomarkers	Major related pathways	Reference
IgAN (*n* = 6), IgAVN (*n* = 5), and controls (*n* = 4), renal tissue	Transcriptomics	ScRNA-seq	HIF1A, ASS1, STAT3, VIM	Hypoxia adaptation, energy metabolism, apoptosis and EMT, kidney repair, urea cycle, renal tubular EMT	(61)
HSP (*n* = 20) and healthy donors (*n* = 4), PBMCs	Transcriptomics	ScRNA-seq	CD40L^+^ MAIT	Activation of immune response, B cell activation, B cell mediated immunity, humoral immune response	(63)
HSPN (*n* = 4) and healthy volunteers (*n* = 4), urine	Proteomics	LC–MS/MS, DIA, ELISA	Integrin beta-1, tenascin	Focal adhesion, cell adhesion molecules, the PI3K-Akt signaling pathway, ECM-receptor interactions	(70)
IgAN (*n* = 4), HSPN (*n* = 4) and healthy volunteers (*n* = 4), urine	Proteomics	LC–MS/MS, DIA, ELISA	A1BG, AFM	Cell adhesion molecules, ECM-receptor interactions, the PI3K-Akt signaling pathway, the complement and coagulation cascades, regulation of actin cytoskeleton, cholesterol metabolism, platelet activation	(71)
HSPN (*n* = 3) and patients after nephrectomy (controls) (*n* = 3), renal tissue	Proteomics	iTRAQ, real-time PCR	CDC42, CTNNB1	Lipid metabolism, adherent junction pathway	(74)
IgAN (*n* = 12), IgAVN (*n* = 12), and healthy transplantation donors (*n* = 5), renal tissue	Proteomics	NLC–MS/MS, immunohistochemistry	Talin 1	Complements and complement-regulating, glomerular damage	(75)
HSP children (*n* = 3) and healthy controls (*n* = 3), PBMCs	Epigenomics	Microarray, quantitative real-time PCR, flow cytometry, ELISA	MiR-1-3p, miR-19b-1-5p, miR-29b-1-5p, miR-483-5p, miR-1246	MAPK, Erb B, Ras, calcium signaling pathway	(93)

IgAN, IgA nephropathy; scRNA-seq, single-cell RNA sequencing; LC-MS/MS, liquid chromatography–tandem mass spectrometry; HSPN, Henoch-Schönlein’s purpura nephritis; iTRAQ, isobaric tags for relative and absolute quantification; nLC-MS/MS, nano-liquid chromatography–tandem mass spectrometry; HIF1A, hypoxia-inducible factor; ASS1, argininosuccinate synthase 1; STAT3, signal transducer and activator of transcription 3; VIM, vimentin; EMT, epithelial–mesenchymal transition; A1BG, alpha-1B glycoprotein; AFM, afamin; PBMC, peripheral blood mononuclear cell.

The deployment of scRNA-seq technology has significantly improved our grasp of the immune mechanisms within the kidneys. This technique facilitates precise identification of gene expression alterations in distinct cell populations and assists in analyzing their overall and specific regulatory patterns. Currently, scRNA-seq studies on HSP mostly utilize blood and kidney tissues to investigate the phenotypes and functions of specific cells. In addition to this, scRNA-seq allows for the analysis of urine cells, including podocytes, PT cells, and collecting duct cells. Evidence suggests that almost every kind of kidney cell can be identified in urine, using either spot urine samples or those collected over 24 h (64). However, 24-h urine samples are prone to cell degradation during storage and are more susceptible to ambient RNA contamination. Compared with kidney tissue, urine samples are easier to obtain. This technology can be applied to urine samples in the future, which is expected to provide broader diagnostic and prognostic value for HSPN.

### 2.3 Proteomics

The proteome refers to the proteins expressed in cells, body fluids or tissues. Proteomics provide detailed information about the proteins in these biological samples and reveal how these proteins interact (33). Compared to genomics and transcriptomics, proteomics may be used to observe the correlation with disease more directly. Proteomics is characterized by integrity, dynamics and stability. Pathogenesis may be better elaborated using the changes in the overall level of proteins in cells or tissues, and specific biomarkers related to diseases may be found. Recent developments in mass spectrometry-based high-throughput techniques and advances in computational analysis have made these applications increasingly feasible and encouraged further research into kidney disease (65). Plasma/serum remains the most important source of samples for proteomics. SELDI-TOF-MS, is a high-throughput protein identification technology that has been automated with high sensitivity and a small sample size (66). Liquid chromatography–tandem mass spectrometry (LC–MS/MS) merges the benefits of mass spectrometry’s strong selectivity and sensitivity with liquid chromatography to effectively separate intricate samples (67). He et al. used highly sensitive nanoscale ultra-performance liquid chromatography-mass spectrometry (nanoLC–MS /MS) to compare serum proteomes and identified a total of 107 differential proteins in the three groups. Four of these proteins were verified using enzyme-linked immunosorbent assay (ELISA). Angiotensinogen (AGT) might serve as a possible indicator for the advancement of IgAV. Furthermore, their functional analysis also showed that hemostasis and Wnt signaling pathways could play a role in the development of IgAV (68) (Table 1). Nano-liquid chromatography–tandem mass spectrometry (nLC–MS/MS) was also used by Demir et al. to explore changes in the plasma proteome. Out of the 418 proteins identified, 5 had reduced expression levels while 15 had elevated expression levels. Furthermore, it was shown that the alternative complement pathway and lectin pathway are essential in IgAV, contributing to its pathogenesis, and the proteins in these pathways might serve as new markers for IgAV. However, proteins like MASP1, CFB, SERPINA5, C9, and GPLD1, which are involved in these vital pathways, were not further examined in this study (69). Although plasma is widely used to study biomarkers for various diseases, proteomics in blood is difficult to detect due to variability after sampling, and many high-abundance proteins, such as immunoglobulin and albumin, which may mask low-abundance proteins.

Urine is rich in proteins and peptides. Compared to blood, proteins and peptides in urine are more stable, resistant to degradation, and easy to transport and store. As a completely non-invasive and repeatable biological sample, urine has played an important role in the early diagnosis of renal injury in recent years and has been gradually applied in proteomics to search for new biomarkers. Fang et al. used the data-independent acquisition (DIA) method to analyze the urine proteins of HSPN patients and healthy controls using LC–MS/MS and validated integrin beta-1 and tenascin, among 125 differentially expressed proteins. The results suggested that tenascin was a marker related to HSPN and may be used for the diagnosis of early renal injury (70) (Table 2). Using the same method, they also found that alpha-1B glycoprotein (A1BG) and afamin (AFM) may be biomarkers for IgAN and HSPN in another study (71) (Table 2). The concentration of protein in normal urine is very low, and high protein abundance may mask low protein abundance. Protein microarray has a good ability to detect low molecular weight proteins, which makes it an ideal method for urinary proteomics research. Marro and colleagues conducted an analysis of urinary protein arrays to assess the amounts of 124 important proteins in children suffering from IgA vasculitis with nephritis (IgAVN). When compared to the group with IgA vasculitis without kidney involvement, the amounts of 20 urinary proteins were found to be higher in IgAVN. The proteins Cripto-1, AGT, sex hormone-binding globulin (SHBG), and B-cell activator (BAFF) displayed the most significant increases and could play a role in the development of IgAVN, though these findings need to be confirmed in a larger study group (72). Although urine proteomics has shown strong predictive ability, the urine protein profile is greatly affected by sex, age, individual differences, exercise, and sampling time. Urine has a lot of urea, salts, and other substances that can easily affect the results of tests. More suitable analytical techniques should be found to improve the detection capability.

Renal proteomics may be used as a complement and extension of serum and urine proteomics, in which renal parenchymal proteomics identifies some proteins that are unexpressed or under-expressed in blood and urine (73). Gao et al. performed the first proteomic analysis in renal tissue from HSPN patients using the isobaric tags for relative and absolute quantification (iTRAQ) method to detect 149 differentially expressed proteins (DEPs). They suggested that CDC42 and CTNNB1 may be candidates for the pathogenesis of HSPN. Functional analysis showed that most of these DEPs were related to lipid metabolism and adhesion junction pathways (74) (Table 2). Kaga et al. were the first to conduct a comparative analysis of IgAN and IgAVN in renal tissues using nLC–MS/MS. They identified 859 proteins. Screening and immunohistochemical verification analysis revealed that the key protein talin 1, crucial for podocyte cytoskeleton stability, was significantly higher in the IgAN-I and IgAVN groups compared to the control. Detecting podocyte lesions early might be possible by measuring talin 1 levels in urine. The research also indicated that IgAN and IgAVN patients share molecular mechanisms responsible for glomerular damage, but the activation of complement in the glomeruli is more pronounced in patients with IgAN. The severity of proteinuria might be connected to the differences in the levels of podocyte-related and basement membrane proteins (75) (Table 2).

In conclusion, proteins in the blood are difficult to detect. In contrast, urine is more stable, has a simple structure, and lacks a self-regulating mechanism. It is of great significance in disease monitoring and prognosis. Although urine can be collected non-invasively, it still lags behind serum in proteomics analysis. Furthermore, although some studies have identified the molecules and signaling pathways involved in HSPN in renal tissue, renal proteomics is limited by the small sample size of renal tissue, the difficulty of obtaining renal tissue, and the heterogeneity of individual tissues. While proteomics methods are important for discovering new biomarkers, they are limited to relative quantification. Machine learning methods based on proteomics have also made great progress in the discovery of biomarkers in recent years (76). Machine learning methods (such as random forest, decision tree, hierarchical clustering, and support vector machine) may be used to extract information features from a large number of proteomic data and establish disease prediction models. In recent years, some studies have employed machine algorithm models to analyze the risk factors related to IgAV-induced kidney damage. The results all indicated that these models performed better in predicting kidney damage caused by IgA vasculitis in children, and had higher prediction accuracy compared to single data analysis (77–79). More importantly, machine learning methods can also be combined with omics technologies to enhance the accuracy of predictive markers, thereby identifying more valuable therapeutic targets (80). However, this method has not yet been adopted in HSPN. Although emerging proteomic techniques are gradually being used to discover new biomarkers, great challenges in clinical application remain. These potential markers must be validated in a large number of subsequent studies and may be combined with AI optimization techniques to improve accuracy.

### 2.4 Metabolomics

Metabolites are metabolic substrates and products that drive the basic functions of cells and are derived from the host, microorganisms, diet, and other exogenous sources. Metabolomics involves studying the small molecules found in bodily fluids, cells, or tissues (81). Compared to genes, transcripts, and proteins, metabolites are less diverse and predict biological processes more accurately (82). The metabolomics of renal diseases has been intensively studied in recent years. The different characteristics of patients with diabetic nephropathy, chronic kidney disease and primary glomerular disease have been discussed, and a variety of metabolites related to disease were discovered using metabolomics techniques (83).

Demir et al. used quadrupole time-of-flight mass spectrometry (Q-TOF LC/MS) to study changes in plasma metabolites in patients with IgAVN. 5-methyltetrahydrofolate, DHAP (18:0), N-acetyl-4-acetylneuraminic acid/N-acetyl-7-O-acetylneuraminic acid, prostaglandin D2/I2, and porphyrogen could be used as predictive markers for IgAV renal injury (84) (Table 1). Sun et al. used ultra-performance liquid chromatography quadrupole time-of-flight tandem mass spectrometry (UPLC-Q-TOF-MS/MS) for serum metabolomics analysis, and metabolic biomarkers were combined with clinical features to predict HSPN. These metabolites, combined with clinical risk factors for HSP kidney injury, may be better predictors of HSPN (85). Boissais et al. were the first to conduct metabolomics research in adult IgAV. They used high-performance liquid chromatography-mass spectrometry to identify four significant metabolites, thymidine, serotonin, L-glutamate, and namely 1-methyladenosine, which have high diagnostic value. However, they did not find any specific characteristics in patients with kidney lesions. This might be related to the differences in the study population and the different analytical detection methods (86). Lipid metabolism might play a role in the development of IgAV. Liu et al. also used LC–MS/MS and UPLC for lipidomic analysis of plasma, and they found that 31 identified lipids were significantly different between IgAV and healthy controls. The increase in TG (16:0/18:1/22:6) + NH4 and PC (32:1) + H and the decrease in PE (21:4)-H significantly increased the risk of IgAV, which predicted IgAV well. PC (38:6) + H was significantly decreased in IgAVN, which may be a potential marker of IgAVN. They suggested that lipid metabolism may influence IgAV pathogenesis via cardiovascular diseases, cell apoptosis, insulin resistance, and inflammation (87) (Table 1). However, none of the metabolites in these studies have been tested in large cohorts.

Urine metabolomics plays a crucial role in finding non-invasive markers that identify slight metabolic variations in certain illnesses or treatment methods. Yu et al. used non-targeted metabolomics technology to identify differentially expressed metabolites in urine, and identified propionylcarnitine and indophenol sulfate as possible predictive biomarkers for HSPN, and might be involved in the oxidative stress response during the HSPN process (88). Zhang et al. used LC-Q/TOF-MS to screen metabolic markers that predicted HSPN progression in serum and urine. They identified 38 differential metabolites in serum and 50 differential metabolites in urine. They screened for two differential metabolites, cis-vaccenic acid and choline, which were found in both serum and urine. ROC analysis showed that the AUCs of choline, cis-vaccenic acid and their combination were 88.69, 79.15 and 92.69%, respectively. The predictive power of the combined panel was better. However, they did not test these factors in large samples (89).

Various factors often affect metabolites in clinical studies, and the results are often difficult to interpret. High variability in urine samples can lead to less accurate metabolite identification. With the advancement of analytical technology and informatics, metabolomics has gradually shifted from biomarkers to disease pathogenesis. Although we identified some metabolites that may be used as biomarkers, the related technology must be optimized to improve its accuracy for clinical application in the future. There are few studies on HSPN metabolomics, and mass spectrometry is primarily used. More potential markers may be found in the future using newer technologies. These techniques may be combined with other omics techniques to provide new insights into disease pathogenesis.

### 2.5 Epigenomics

Epigenetics is a heritable modification affecting gene expression and regulation that does not involve changes in DNA sequence and is influenced by genetic and external factors. It includes DNA methylation, histone modification (such as methylation, acetylation or ubiquitination) and non-coding RNA (ncRNA) interference (90). Luo and colleagues discovered higher amounts of H3 acetylation and H3K4 methylation in the PBMCs of HSP patients who had kidney damage, which were linked to the seriousness of the disease. Differential expression of histone modifier genes may lead to a shift in the Th1/Th2 balance toward Th2 immunity. The promoter and enhancer regions of IL-4 in CD4 + T cells of HSP patients showed significantly increased H3 acetylation levels and H3K4 methylation levels, which may lead to impaired IL-4 production and affect Th2-mediated immune response (91).

NcRNAs do not directly encode proteins, but participate in the translation process of proteins and mostly play a regulatory role during and after protein transcription (92). Li et al. used microRNA (miRNA) microarrays to analyze miRNA levels in PBMCs of children with HSP and healthy children, and found that 27 miRNAs were downregulated, and nine miRNAs were upregulated. KEGG enrichment showed that the ErbB, MAPK, Ras and calcium signaling pathways were the main pathways involved. Quantitative real-time PCR measurements showed that miR-29b-1-5p, miR-19b-1-5p and miR-1-3p were up-regulated, and miR-483-5p and miR-1246 were downregulated in PBMCs of HSP children compared to healthy controls. These miRNAs might be able to tell apart individuals with HSP from those who are healthy. They also found that these miRNAs were associated with serum IL-6, IgA or Th17/Treg, and may be involved in the pathogenesis of HSP (93) (Table 2). LncRNAs interfere with gene transcription, regulate cell differentiation, and participate in stress and inflammatory responses (94). Pang et al. identified 820 long non-coding RNAs (lncRNAs) and 3,557 mRNAs differentially expressed in HSPN and healthy children using high-throughput sequencing in the peripheral blood of a small cohort. Among the six lncRNAs verified, ENST00000571370, ENST00000378432, UC010QNA.2 and UC001KFC1–1 were downregulated in HSPN patients and were related to the p53 signaling pathway and apoptosis-related genes. These elements could play a key role in the development of HSPN (95) (Table 1). A novel post-transcriptional regulatory mechanism, the competitive endogenous RNA (ceRNA) theory, suggests that lncRNAs and mRNAs form regulatory network interactions via miRNAs (96). Huang et al. constructed an lncRNA-miRNA-mRNA regulatory network related to immunity and apoptosis in children with HSPN. 11 lncRNAs were dysregulated. FGD5-AS1, DLEU2, SCARNA9, SNHG3, LINC00152, TUG1 and GAS5 were upregulated, and DISC1-IT1, PVT1, SNHG1 and NEAT1 were downregulated. They also validated these lncRNAs in clinical samples, which suggested novel biomarkers for the diagnosis and treatment of HSPN (97). Although this study showed that epigenomics provided a direction for the screening of important biomarkers in HSPN, RNA is unstable and easily degraded, and challenges remain for future application of high-throughput techniques. Current studies on HSPN have been confined to peripheral blood or PBMCs. Therefore, new epigenetic modification genes may be identified in urine or tissues in the future.

## 3 Conclusion

**Figure 1:**
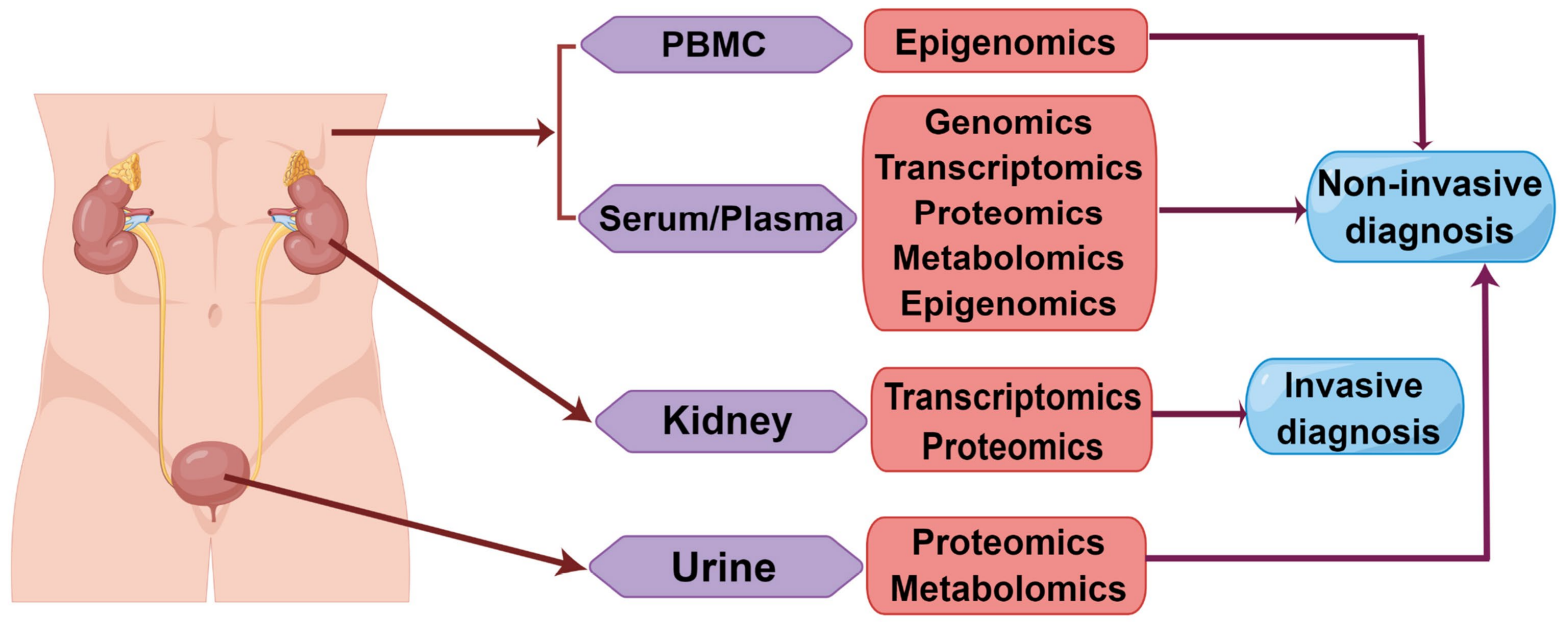
Overview of the use of omics to screen biomarkers for early diagnosis of HSPN. HSPN can alter the expression of several molecules. These molecules can be released into the blood, urine, or deposited in kidney tissue, where they can be measured. Omics is an important method for screening potential biomarkers. PBMC: peripheral blood mononuclear cell (By Figdraw).

Although HSPN is a common autoimmune disease, the ability to diagnose early kidney injury is limited. Current omics studies explored some potential noninvasive markers for early diagnosis. These markers are readily available and safer, which makes them ideal for clinical application. Tables 1, 2 summarize some potential biomarkers that have been discovered in the blood, urine, kidney tissues and cells of HSPN patients and have been verified, as well as list the important pathways related to the pathogenesis of HSPN. However, most of these markers have only been verified in small samples, and their clinical application remains challenging. A large number of multicenter, large-sample validation studies are needed to evaluate its sensitivity and specificity to determine its value in early diagnosis. Among these omics technologies, DNA appears to be static and uniform throughout the body, while RNA, proteins and metabolites are dynamic. Genomics has shown that genetic elements play a crucial role in the development of this illness, particularly the link to the HLA II area, which can enhance our understanding of how HSPN develops. Omics techniques have provided us with an in-depth understanding of the pathogenesis of HSPN, such as the use of GWAS to identify potential sites associated with abnormal glycosylation (32). Transcriptomics, especially scRNA-seq technology, is gradually being applied in HSPN to assess the dynamic expression of genes, discover more valuable functional pathways, and thereby clarify the significant roles of these important genes in the diagnosis and treatment of HSPN. Moreover, urine, as a non-invasive sample, requires further exploration of its RNA-seq technology in HSPN. Although proteomics and metabolomics are closer to disease phenotypes, being able to provide information about the biological status at specific times and locations, compared to genomics and transcriptomics, the obtained data is complex and difficult to analyze, making it impossible to infer the causal relationship between these markers and the disease. Therefore, it is difficult to analyze their role in the pathogenesis of HSPN. Given this, artificial intelligence and machine learning technologies need to be further explored. Cao et al. predicted kidney injury in children with HSP based on the XGBoost model. The indices based on the algorithm included NAG, RBP and IgA. The evaluation of predictive performance showed that the area under the curve of the model was 0.895 in the training set and 0.870 in the test set, with good sensitivity and specificity (98). The construction of the prediction model effectively reduced the overfitting problem and improved the efficiency of the algorithm. However, this model only studied laboratory indicators and was not combined with omics techniques to explore new markers. Artificial intelligence techniques, such as machine learning, may be combined with omics techniques in the future to optimize the screening and analysis of biomarkers. We investigated the potential of molecular markers from the blood, urine, tissues, and cells of HSPN patients using omics analysis (Figure 1). This study contributes to early diagnosis, the exploration of the molecular mechanism of the disease and helps determine the prognosis of the disease and search for new therapeutic targets.
